# Evaluation of the Efficacy of Anthelmintic Drugs Against *Trichinella spiralis* Larvae

**DOI:** 10.3390/antibiotics15020215

**Published:** 2026-02-16

**Authors:** Soon-Ok Lee, Su In Heo, Hyeon-Woo Nam, Ji-Hyun Lee, Ki Back Chu, Gi-Ja Lee, Tong In Oh, Sung Soo Kim, Fu-Shi Quan

**Affiliations:** 1 Department of Medical Zoology, School of Medicine, Kyung Hee University, Seoul 02447, Republic of Korea; 2 Department of Biomedical Science, Graduate School, Kyung Hee University, Seoul 02447, Republic of Korea; 3 Department of Parasitology, Inje University College of Medicine, Busan 47392, Republic of Korea; 4 Department of Infectious Disease and Malaria, Paik Institute of Clinical Research, Inje University, Busan 47391, Republic of Korea; 5 Department of Biomedical Engineering, College of Medicine, Kyung Hee University, Seoul 02447, Republic of Korea; 6 Medical Research Center for Bioreaction to Reactive Oxygen Species and Biomedical Science Institute School of Medicine, Graduate School, Kyung Hee University, Seoul 02447, Republic of Korea

**Keywords:** *Trichinella spiralis* larvae, tribendimidine, tetrazolium salt, anthelmintics

## Abstract

**Background**: Albendazole, mebendazole, and ivermectin are effective against adult *Trichinella spiralis* but show limited efficacy against encapsulated muscle stage larvae. This limitation highlights the need for improved experimental approaches to evaluate anthelmintic activity at this stage and to identify alternative therapeutic candidates. **Methods**: Seven antiparasitic drugs, albendazole (ABZ), miltefosine (MLT), ivermectin (IVM), tribendimidine (TBD), praziquantel (PZQ), artesunate (ART), and mefloquine (MEQ), were evaluated for in vitro activity against *T. spiralis* muscle larvae. Larval viability was quantified using a tetrazolium salt XTT assay to determine IC_50_ values and compare with microscopic assessments. Based on in vitro activity, TBD was selected for in vivo evaluation in a mouse model, where efficacy was assessed by muscle larval burden and histopathological changes. **Results**: TBD, MEQ, IVM, and ABZ exhibited measurable in vitro efficacies against *T. spiralis* larvae, with TBD showing the lowest IC_50_ value at 135.2 μM. XTT formazan absorbance correlated strongly with larval number and incubation time. In vivo, TBD treatment significantly reduced larval burdens in diaphragm and gastrocnemius muscles and was associated with reduced collagen capsule thickness, inflammation, and fibrosis compared with ABZ-treated controls. **Conclusions**: This study validated an assay for quantitative evaluation of *T. spiralis* muscle larvae and demonstrates robust in vitro and in vivo activity of TBD against this stage.

## 1. Introduction

Trichinellosis is a zoonotic parasitic disease caused by *Trichinella spiralis*, a nematode belonging to the genus Trichinella, and represents a public health concern for both humans and animals [1,2]. It is estimated that up to 11 million people worldwide are infected with *Trichinella* spp., and the disease remains endemic or recurrent in several regions of the world [3,4]. Human infection occurs through the consumption of raw or undercooked meat containing infective larvae and can result in a spectrum of clinical manifestations, ranging from mild gastrointestinal symptoms to systemic complications such as myalgia and fever [5,6].

Benzimidazole derivatives, including albendazole and mebendazole, are currently the primary drugs used for the treatment of human trichinellosis. However, their clinical efficacy is limited by poor water solubility and variable bioavailability [2]. In addition, these compounds show reduced activity against encysted muscle larvae, a stage that is particularly refractory to chemotherapy [7,8]. The use of benzimidazoles is also contraindicated in pregnant women and young children because of potential safety concerns [9]. Furthermore, reports describing adverse effects and possible long-term toxicological risks have highlighted the need for continued evaluation of alternative therapeutic options [10]. Consequently, the development of novel or repurposed drugs with improved efficacy and safety profiles remains an important objective in trichinellosis control.

Current methods for evaluating the in vitro efficacy of drugs against *T. spiralis* typically rely on culturing specific developmental stages of the parasite and assessing drug-induced effects based on viability, motility, or morphological alterations [11,12]. These approaches are labor-intensive, require experienced personnel, and are inherently subjective. Moreover, many anthelmintic candidates have historically been identified through in vivo screening in animal models, a strategy that is time-consuming, low-throughput, and associated with substantial animal use [13,14]. These limitations complicate the quantitative evaluation of drug effects and hinder efficient prioritization of candidate compounds for further testing, supporting the need for cost-effective and higher-throughput in vitro approaches that provide more objective readouts.

Tetrazolium salt-based assays offer a colorimetric approach for quantifying metabolic activity as a surrogate marker of viability. The XTT assay employs a water-soluble tetrazolium salt that is reduced by metabolically active cells to produce a measurable colorimetric signal. This method has been applied to a range of organisms, including bacteria [14], fungi [15], protozoan parasites [16], and helminths [17,18]. However, the performance characteristics of XTT-based metabolic readouts for assessing *T. spiralis* larval viability in drug testing have not been well defined. To address this need, the present study evaluated an XTT-based colorimetric approach for quantifying larval viability during in vitro drug exposure and compared these results with microscopic assessment. We further examined the in vivo relevance of selected findings using a murine infection model. Using a panel of compounds with reported or putative antiparasitic activity, we assessed in vitro effects on larval viability and identified candidates for follow-up evaluation.

## 2. Results

**Figure 1:**
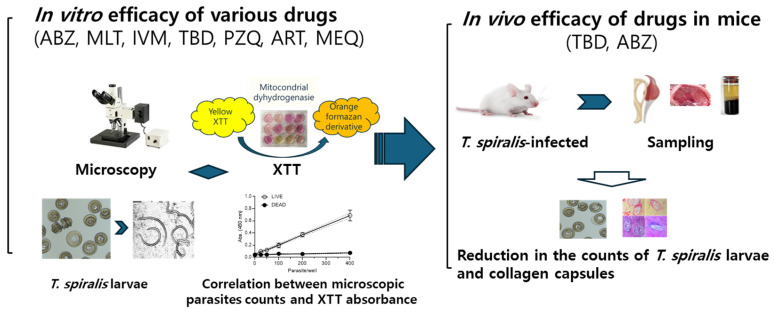
Schematic overview of the experimental workflow for evaluating anthelmintics’ efficacy against *T. spiralis*. *T. spiralis* muscle larvae were exposed in vitro to seven anthelmintics for 24 h to screen drug efficacy. Larval viability and IC_50_ were quantified using XTT assay and compared with microscopic observations. TBD, which exhibited the highest in vitro efficacy, was selected for in vivo evaluation. Therapeutic efficacy in mice was assessed by reductions in muscle larval burden and by histopathological changes, including collagen capsule thickness, inflammation, and fibrosis.

*Experimental design to evaluate anthelmintic efficacy*. An overview of the experimental design is shown in Figure 1. The in vitro efficacies of the seven drugs were evaluated against *T. spiralis* muscle larvae. Larval viability was assessed by microscopic observation, and the relationship between microscopically determined parasite counts and XTT assay absorbance was examined. The in vivo efficacies of TBD and ABZ were evaluated in infected mice by quantifying larval burdens in the diaphragm and gastrocnemius muscles. Parasite capsule thickness, capsule degeneration, and inflammatory changes in the gastrocnemius muscle were assessed following drug treatment.

**Figure 2:**
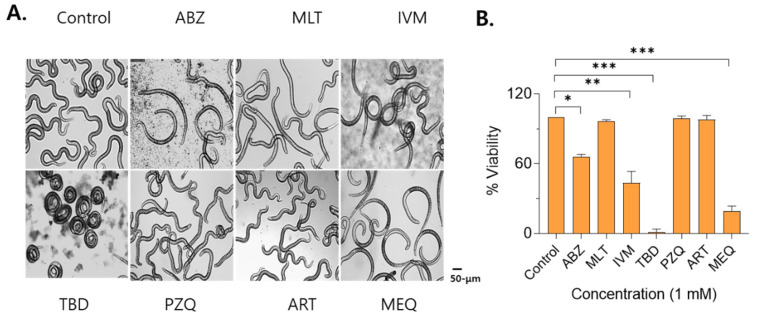
In vitro effects of various anthelmintics on *T. spiralis* larvae. One hundred larvae were incubated with 1 mM of each drug (ABZ, MLT, IVM.TBD, PZQ, ART, MEQ) for 24 h. Representative microscopic images showing drug-induced morphological changes (**A**). Larval survival rates determined by changes in motility (**B**). All images were acquired at 200× magnification. Scale bar, 50 μm. Statistical significance was indicated using asterisks (* *p* < 0.05, ** *p* < 0.01, *** *p* < 0.001).

*In vitro anti-T. spiralis larval activity.* For the primary single-dose screen, larvae were incubated with each compound at 1 mM for 24 h to identify candidates with measurable anthelmintic activity against T. spiralis. Exposure to TBD resulted in a marked larval contraction, with near-complete larval death. Treatment with IVM reduced larval motility and induced partial contraction, with a mortality rate of 56.7%. Following ABZ or MEQ treatment, most of the larvae lost axial rigidity and exhibited a flaccid, immotile morphology, with mortality rates of 34% and 80.7%, respectively. In contrast, larvae treated with ART, MLT, or PZQ exhibited motility patterns comparable to those of untreated controls, and no larval death was observed (Figure 2A,B).

**Figure 3:**
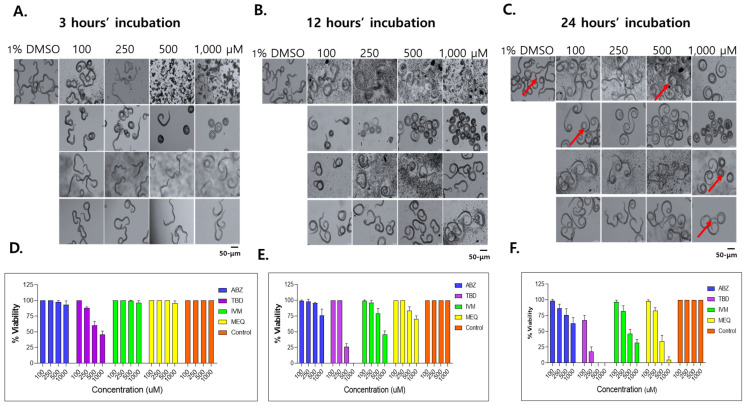
Dose- and time-dependent effects of selected anthelmintics on *T. spiralis* larvae. For in vitro survival assays, 25 to 400 muscle larvae were incubated with ABZ, IVM, MEQ, or TBD at concentrations of 100, 250, 500, and 1000 µM for 3, 12, and 24 h (**A**–**C**). Drug efficacy was assessed by microscopic evaluation of larval morphology and survival (**D**–**F**). Larvae were classified as viable when spontaneous movement or migration was observed, including markedly reduced motility, and as non-viable when no movement was detected even after gentle mechanical stimulation. Survival percent was calculated as the number of viable larvae divided by the total number of larvae assessed, multiplied by 100. Red arrows indicate non-viable larvae after 24 h of drug exposure. Representative images were acquired at 200× magnification. Scale bar, 50 μm.

*Dose- and time-dependent effects of selected drugs on T. spiralis larval viability*. Dose- and time-dependent effects of ABZ, IVM, MEQ, and TBD on muscle larva were further evaluated based on larval morphology and motility. After 3 h of exposure, larvae treated with 100 μM TBD exhibited control-like movement, whereas treatment with 250 μM resulted in reduced motility and larval contraction, with approximately 40–50% of larvae exhibiting no movement. No notable morphological changes or mortality were observed at this time point in the ABZ, IVM, or MEQ groups (Figure 3A,D). After 12 h of exposure, treatment with 500 to 1000 μM TBD induced pronounced larval contraction, with complete immobilization observed in 74% and 100% of larvae, respectively. IVM induced larval contraction at all tested concentrations, with mortality rates of 21.3% and 54.7% at 500 to 1000 μM, respectively. MEQ treatment induced excessive larval movement, followed by relaxation and partial mortality at higher concentrations. In contrast, ABZ induced minimal morphological changes and limited larval death (Figure 3B,E).

After 24 h of exposure, larval motility was reduced in all treatment groups, whereas active movement persisted in control larvae (Figure 3A–C). At concentrations of 500 to 1000 μM, larval mortality ranged from 54–68% for IVM, 65.3–95.3% for MEQ, and 24–37.3% for ABZ. In the TBD-treated groups, mortality reached 32.0% and 82.0% at 100 and 250 μM, respectively, and complete larval death was observed at 500 μM (Figure 3D–F). Morphological observations suggested that larval death in the TBD-, IVM-, and ABZ-treated groups was associated with sustained muscular contraction, whereas MEQ-induced larval death was accompanied by excessive motility followed by apparent exhaustion.

**Figure 4:**
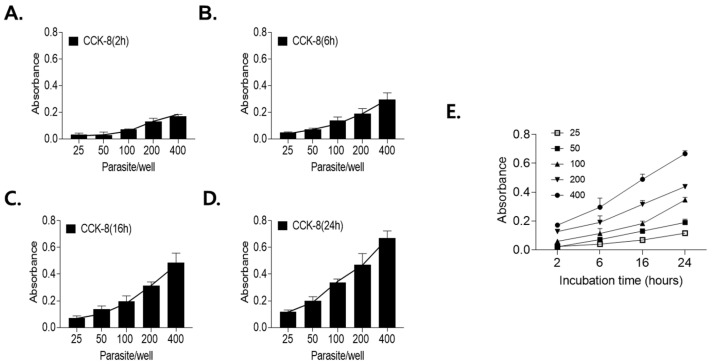
Correlation between XTT formazan absorbance and the number of viable *T. spiralis* larvae. Formazan absorbance increased proportionally with incubation time at two larval densities, with 24 h identified as the optimal incubation period (**A**–**D**). Assay sensitivity was evaluated using 25, 50, 100, 200, and 400 larvae per well. Linear regression analysis revealed a strong linear relationship between formazan absorbance and larval concentration (**E**).

*Correlation between parasite number and XTT absorbance.* To optimize the XTT-based viability assay, the effects of the parasite number and incubation time on assay performance were evaluated. A positive correlation between XTT absorbance and incubation time was observed at both tested larval concentrations (Figure 4A–D). An incubation time of 24 h was selected as optimal for distinguishing differences in larval numbers. Assay sensitivity was subsequently evaluated using 25, 50, 100, 200, and 400 larvae per well. Linear regression analysis demonstrated a strong linear relationship between formazan absorbance and larval number (Figure 4E). Wells containing more than 100 larvae produced absorbance values sufficient to reliably distinguish live larvae from heat-killed controls at all incubation times. Accordingly, subsequent assays were performed using 100 larvae per well in a 24-well plate format with a 24 h incubation period.

**Figure 5:**
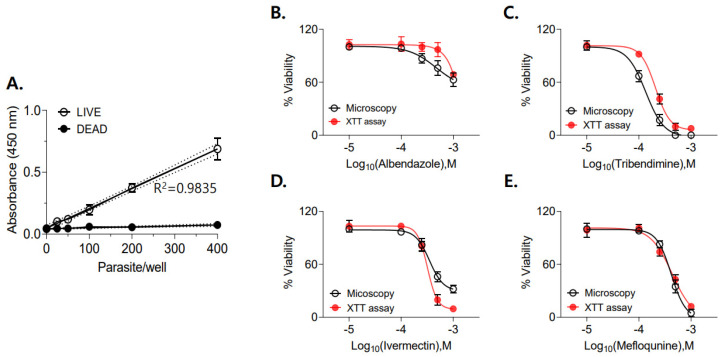
Comparison of drug sensitivity determined by microscopic observation and XTT viability assay. To determine whether IC_50_ values could be calculated using XTT-based viability analysis, four active compounds were tested at concentrations of 100, 250, 500, and 1000 µM for 24 h (**A**). Heat-killed larvae were used as negative controls, and larvae treated with 0.1% DMSO served as positive controls. Larval viability was quantified using XTT absorbance and compared with microscopic assessments (**B**–**E**). Wells containing drugs alone were included to exclude potential interference with the colorimetric assay.

*Determination of IC*_50_ *values using the XTT assay.* The sensitivity of the XTT assay to detect partial reductions in larval viability was evaluated by preparing mixtures containing defined ratios of live and heat-killed larvae. Formazan absorbance increased proportionally with the fraction of live larvae, while heat-killed larvae consistently yielded absorbance values below 0.1 (Figure 5A). Drug susceptibility assays were then performed to compare IC_50_ values determined by XTT analysis with those obtained by microscopic assessment. The IC_50_ values for ABZ, IVM, MEQ, and TBD were calculated based on parasite viability derived from absorbance values and compared with microscopic estimates (Figure 5B–E; Table 1). TBD and IVM exhibited lower IC_50_ values than ABZ in both microscopic and XTT-based analyses, indicating greater larvicidal potency against *T. spiralis* muscle larvae under the tested conditions.

**Figure 6:**
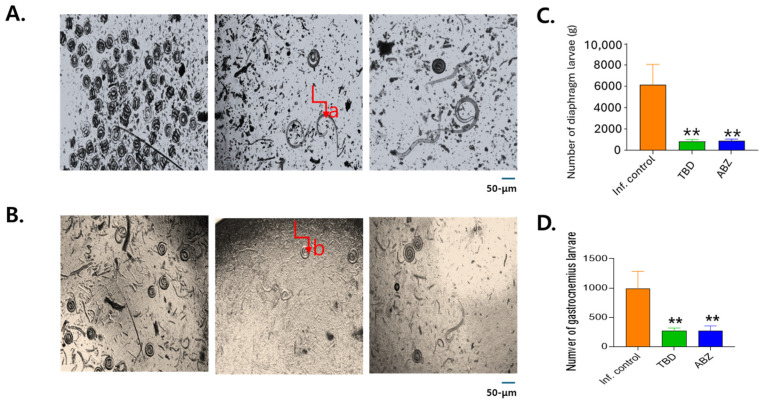
In vivo efficacy of TBD and ABZ against muscle stage *T. spiralis* larvae. Infected mice were randomly assigned to untreated, TBD, or ABZ treatment groups. Treatments were administered for 3 consecutive days starting at 35 dpi. Mice were sacrificed 10 days after treatment. Diaphragm and gastrocnemius were digested with pepsin–HCl, and larval burden was expressed as the number of larvae per gram of tissue (**A**–**D**). a: Dead larva. b: Live larva. Data are presented as mean ± SD, and asterisks indicate significant difference relative to the infection control group (** *p* < 0.01).

*In vivo efficacy of TBD against T. spiralis muscle larvae.* The in vivo efficacy of TBD was evaluated in comparison with ABZ in *T. spiralis*-infected mice. Morphologically damaged or dead larvae were occasionally observed in treated tissues (Figure 6A,B). Larval burdens were quantified following artificial digestion of diaphragm and gastrocnemius muscles. In the untreated infection control group, larval burdens averaged 6169 ± 1921 and 989  ±  295.7 larvae per gram in the diaphragm and gastrocnemius muscles, respectively (Table 2; Figure 6C,D). Both treatment regimens significantly reduced larval burdens in the diaphragm and gastrocnemius muscles. In the TBD-treated group (300 mg/kg), larval counts were reduced to 799 ± 199.4 (87.0%), 265.0 ± 53.2 (73.2%), respectively. Similarly, ABZ treatment (150 mg/kg) reduced larval counts to 844 ± 184.6 (86.3%), 259.5 ± 77.5 (73.8%), respectively. Both TBD- and ABZ-induced reductions were statistically significant compared with the infection control group (** *p* < 0.01). No statistically significant differences were observed between the TBD- and ABZ-treated groups.

**Figure 7:**
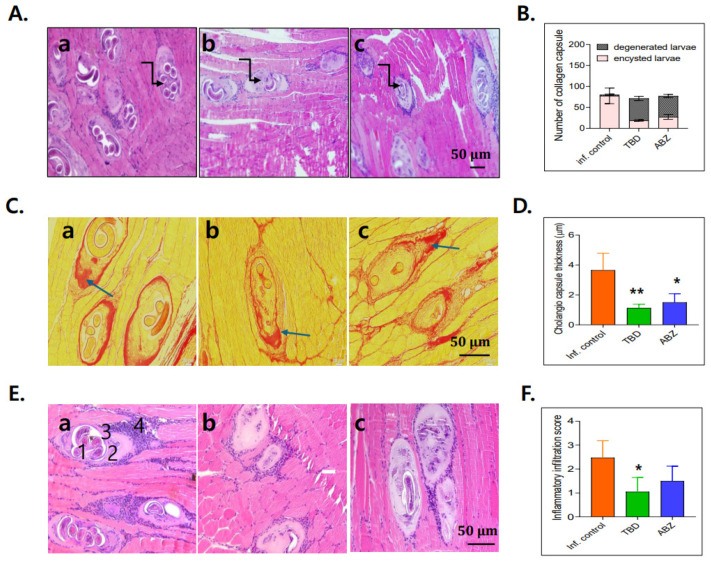
Histopathological changes in muscle tissue following anthelmintic treatment. (**A**): H&E-stained muscle sections. (**a**) Numerous viable larvae were observed in untreated infected controls; (**b**) albendazole treatment reduced the number of larvae, and degenerative forms were present; (**c**) TBD treatment resulted in very few larvae, most of which showed degenerative changes. Images were captured at 100× and 200× magnification. (**B**): Quantification of encysted and degenerative larvae. (**C**): PSR-stained muscle sections showing collagen capsule formation. (**a**) Thick, continuous fibrous capsules were observed in untreated controls; (**b**) thin capsules with focal disruption appeared after albendazole treatment; (**c**) markedly thin capsules with extensive local disruption were seen after TBD treatment. Images were obtained at 200× magnification. (**D**): Measurement of fibrous capsule thickness. (**E**): Inflammatory infiltration. (**a**) Severe inflammatory infiltration was present in untreated infected controls; (**b**) mild infiltration was observed after albendazole treatment; (**c**) TBD treatment showed a small number of larvae, including degenerative forms, accompanied by moderate inflammatory infiltration. (**F**): Inflammatory cell infiltration scores. Data are presented as mean ± SD, and asterisks indicate significant difference relative to the infection control group (* *p* < 0.05, ** *p* < 0.01).

*Histopathological analysis.* Muscle sections from untreated infected mice exhibited numerous viable *T. spiralis* larvae enclosed within intact collagenous capsules (Figure 7A(a)). In contrast, treated groups exhibited degenerative capsules accompanied by inflammatory cell infiltration and degenerated larvae within the capsules (Figure 7A(b,c)). Capsule thickness was evaluated using PSR staining. Degenerative capsules in all treatment groups were significantly thinner than normal capsules in untreated controls. Moreover, capsule thickness in the TBD-treated group was significantly reduced compared with that in the ABZ-treated group (Figure 7C,D). Untreated infected muscle exhibited extensive degeneration, with viable larva surrounded by collagen capsule and inflammatory infiltrates composed predominantly of lymphocytes, plasma cells, eosinophils, and histiocytes. These pathological features were most prominent at both poles of the capsule and were characterized by the presence of (1) live larvae, (2) nurse cells, (3) collagen capsules, and (4) associated inflammatory cells (Figure 7E(a)). In contrast, muscle sections from TBD- and ABZ-treated mice exhibited reduced inflammatory infiltration and increased replacement of degenerated larvae by homogenous eosinophilic material (Figure 7E(b,c)). Quantitative analysis demonstrated a significant reduction in inflammatory cell density in the TBD-treated group (Figure 7F).

## 3. Discussion

A major limitation in advancing therapies against muscle-stage *T*. *spiralis* infection is the reduced efficacy of existing anthelmintics combined with the lack of quantitative and reproducible experimental tools for evaluating this stage. Benzimidazole derivatives are most effective during the intestinal phase of infection, yet their efficacy declines once *T*. *spiralis* larvae establish within muscle tissue [19,20]. Reduced drug accessibility, altered parasite physiology, and encapsulation likely contribute to the limited therapeutic impact at this stage [21,22]. Although early treatment may prevent larval establishment, most human infections are diagnosed after muscle invasion, constraining the clinical relevance of current interventions [20]. Attempts to overcome these limitations through dose escalation are further restricted by poor solubility, variable bioavailability, and dose-associated toxicity [19]. Together, these factors support the need for experimental systems that can reliably assess drug effects on muscle-stage larvae and inform therapeutic development. In this study, we evaluated an XTT-based metabolic readout as a quantitative in vitro approach for assessing muscle larval viability and compared the activity of selected non-benzimidazole compounds against this developmental stage.

Our results indicate that muscle stage larvae are not metabolically inert despite their encysted state. The consistent reduction of XTT by these larvae indicates sustained metabolic activity sufficient to support redox-based viability measurements [23]. This finding is consistent with prior histological and physiological studies indicating that muscle larvae actively maintain the nurse cell complex and engage in parasite–host metabolic exchange [24,25,26]. Persistence of metabolic function within a structurally protected niche likely contributes to the long-recognized discrepancy between in vitro drug sensitivity and incomplete in vivo clearance observed for many anthelmintics [6]. From a methodological perspective, the XTT readout provides an objective measure that can complement microscopy in drug testing. XTT-based measurements can support quantitative comparisons across conditions, particularly when microscopy yields intermediate phenotypes that are difficult to classify. Traditional in vitro evaluation of *T*. *spiralis* relies heavily on motility and morphological scoring, approaches that are inherently subjective, labor-intensive, and poorly suited for comparative or high-throughput analysis [6,19]. By shifting the experimental focus from simple motility loss to quantitative viability, the XTT-based platform enables more nuanced comparisons of drug efficacy and provides a rational basis for dose–response analysis. Accordingly, our results demonstrated strong correlations between larval number, incubation time, and formazan production, and showed agreement with microscopic assessment, supporting metabolic activity as a practical and experimentally tractable endpoint for comparing drug effects on muscle-stage larvae under our experimental conditions.

Application of this assay revealed that chemically and mechanistically distinct anthelmintics converge on a limited set of physiological failure modes in muscle larvae. In this study, ABZ, IVM, and TBD predominantly induced sustained contraction and immobilization, whereas MEQ exposure was associated with increased motility followed by loss of movement. These patterns are consistent with drug-class-dependent disruption of larval function, although the present study does not establish specific mechanisms [14,27]. Notably, IVM has been reported to exhibit activity against adult and migrating *T*. *spiralis* larvae while showing reduced efficacy against encysted diaphragm larvae in vivo, highlighting the stage- and tissue-dependent nature of drug susceptibility [27]. Within this framework, TBD showed strong in vitro effects in our assay, consistent with cholinergic neuromuscular pathways as plausible targets in muscle-stage larvae. TBD acts as a nicotinic acetylcholine receptor agonist, a mechanism distinct from the microtubule inhibition mediated by benzimidazoles [28]. Its strong in vitro metabolic suppression suggests that disruption of cholinergic neuromuscular signaling can partially overcome the protective constraints imposed by the muscle niche. However, the absence of clear superiority over ABZ reinforces the principle that in vitro potency alone is insufficient to predict therapeutic dominance once host pharmacokinetics, tissue distribution, and parasite encapsulation are considered. Consistent with this interpretation, both TBD and ABZ significantly reduced larval burdens in muscle tissues, yet neither achieved complete elimination.

## 4. Materials and Methods

### 4.1. Preparation of Antiparasitic Drugs

Seven antiparasitic drugs were evaluated in this study: albendazole (ABZ; Sigma-Aldrich, St. Louis, MO, USA), artesunate (ART; Cayman Chemical Company, Ann Arbor, MI, USA), ivermectin (IVM; Cayman Chemical Company, Ann Arbor, MI, USA), mefloquine (MEQ; MedChemExpress, Monmouth Junction, NJ, USA), miltefosin (MLT; Cayman Chemical Company, Ann Arbor, MI, USA), praziquantel (PZQ; Cayman Chemical Company, Ann Arbor, MI, USA), and tribendimidine (TBD; MedChemExpress, Monmouth Junction, NJ, USA). All compounds were dissolved in dimethyl sulfoxide (DMSO) to prepare stock solutions at a concentration of 100 mg/mL and stored at −20 °C until use.

### 4.2. In Vitro Anthelmintic Activity Against T. spiralis Larvae

In vitro drug susceptibility assays were performed using sterile 24-well tissue culture plates (SoCal Biomed, Newport Beach, CA, USA). Muscle larvae of *T. spiralis* were maintained in RPMI-1640 medium supplemented with 10% fetal serum, 200 U/mL penicillin, and 200 μg/mL streptomycin. Based on a preliminary range-finding experiment with ABZ (0.5, 1, and 5 mM), 1 mM was selected as a standardized screening concentration for the primary 24 h screen against *T. spiralis* larvae. Initially, 100 muscle larvae in each well were incubated with 1 mM of each drug (ABZ, ART, IVM, MEQ, MLT, PZQ, TBD) for 24 h, with a vehicle control group to screen for efficacious anthelmintic drugs. Based on these preliminary results, subsequent dose- and time-response experiments were conducted using ABZ, IVM, MEQ, and TBD. For these assays, 25, 50, 100, 200 and 400 muscle larvae were incubated with 100, 250, 500, and 1000 µM of each compound for 3, 12, or 24 h. Larval viability was assessed based on motility and morphological integrity under light microscopy. Larvae were considered viable if any spontaneous movement or migration was observed, even when motility was markedly reduced. A total of three independent experiments were performed, each with technical triplicates per condition. Survival rates were calculated using the equation described below:

(1) $$\%Viability=\frac{Number\;of\;live\;larvae}{\left[Number\;of\;live\;larvae\;+\;Number\;of\;dead\;larvae\right]}\times 100\%$$

### 4.3. Parasite Viability Assessment Using XTT Assay

Parasite metabolic viability was evaluated using the tetrazolium salt sodium-2,3-bis-(2-methoxy-4-nitro-5-sulfophenyl)-2H-tetrazolium-5-carboxanylide (XTT; Sigma-Aldrich), with minor modification to a previously described protocol [29]. Muscle larvae were incubated at 37 °C for 2, 6, 16, or 24 h prior to XTT analysis. For each assay, 40 μL of XTT solution was added to each well of a 24-well plate containing 100 μL of larval suspension. Absorbance was measured using six larval densities (25, 50, 100, 200, 400 larvae per well). Optical density was recorded at 450 nm using a SpectraMax 5 microplate reader (Molecular Devices, San Jose, CA, USA). A total of three independent experiments were performed, each with technical triplicates per condition. Parasite viability was calculated using the following equation:

(2) $$\%Viability=\frac{\left[Sample-Positive\;control\right]}{\left[Negative\;control\;-\;Positive\;control\right]}\times 100\%$$

“Sample” refers to the mean absorbance measured in wells containing the drug-treated larvae, “negative control” refers to the mean absorbance measured in heat-killed larvae which were incubated at 65 °C for 10 min, and “positive control” refers to the mean absorbance of untreated, viable parasites. Microscopic examination was performed in parallel to confirm parasite viability and to verify that XTT exposure did not induce structural damage or artificial mortality.

### 4.4. IC_50_ Determination Using Microscopic and XTT-Based Analyses

To assess whether IC_50_ values could be reliably estimated using XTT-based viability measurement, four compounds demonstrating in vitro activity against *T. spiralis* larvae were selected for dose–response analysis. Stock solutions were prepared in DMSO and serially diluted in RPMI-1640 medium. Larvae were incubated with each compound at concentrations of 100, 250, 500, or 1000 µM for 24 h, followed by XTT viability analysis as described above. Parasite viability was calculated using normalized absorbance values relative to the untreated controls, while heat-killed larvae served as negative controls. To exclude potential interference of drug-associated coloration with XTT absorbance readings, wells containing drug solutions without larvae were included and used for background calibration. XTT-based viability results were directly compared with microscopic viability assessments to evaluate concordance between the two methods.

### 4.5. Evaluation of Drug Efficacy in T. spiralis-Infected Mice

Female BALB/c mice (6–8 weeks old) were purchased from Young Bio (Seongnam, Republic of Korea) and maintained under standard laboratory conditions with free access to commercial rodent chow and water. All animal procedures were approved by the Kyung Hee University Animal Ethics Committee (IACUC approval number: KHUASP (SE)-18-050). *T. spiralis* parasites were maintained through serial passage in BALB/c mice. A total of 16 mice (n = 4 per group) were randomly divided into four groups: an uninfected control group, an infected control group, an ABZ-treated group, and a TBD-treated group. Mice were orally infected with 300 *T. spiralis* larvae, and at 35 days post-infection (dpi), animals were treated with TBD (300 mg/kg) once daily for three consecutive days. ABZ-treated mice (150 mg/kg) served as a positive treatment control, while infected but untreated mice served as negative controls. Body weight and clinical signs were monitored daily throughout the in vivo study in accordance with the approved animal protocol. Ten days after the final dose, all mice were anesthetized and euthanized with CO_2_ inhalation followed by cervical dislocation. Mice were sacrificed 10 days after completion of treatment. Tissues samples were acquired from predilection sites including diaphragm and gastrocnemius. Collected samples were weighed and digested in 1% pepsin-HCl at 37 °C for 5 h. Larval burdens were determined using a sedimentation technique and are expressed as the mean number of larvae per gram of muscle tissue. Diaphragm larvae were additionally quantified by direct microscopic examination following careful dissection [30].

### 4.6. Histopathological Analysis

Gastrocnemius muscle tissues were collected for histopathological evaluation. Samples were fixed in 10% neutral-buffered formalin, washed with water for 12 h, dehydrated through graded alcohols, cleared with xylene, and embedded in paraffin. Five-micrometer sections were prepared using a microtome and stained with hematoxylin and eosin (H&E) or picrosirius red (PSR). Histological sections were examined under light microscopy to assess larval density, inflammatory responses, and fibrosis. Larval density was scored at ×100 magnification as follows: +1 (<5 larvae per field), +2 (5–10 larvae per field), and +3 (>10 larvae per field). Inflammatory responses surrounding infected muscle fibers were scored as mild (+1), moderate (+2), or severe (+3). For each sample, ten low-magnification (×10) fields were examined, and mean scores were calculated according to previously described criteria [21]. Fibrosis and collagen capsule thickness were quantified using ImageJ software (1.53q). Three randomly selected images per slide were captured at ×400 magnification, and capsule thickness ratios were calculated to determine the extent of fibrosis.

### 4.7. Statistical Analysis

Statistical analyses were performed using GraphPad Prism version 7. Two-way repeated-measures analysis of variance (ANOVA) (concentration × time), followed by Tukey’s multiple comparisons test, was applied where appropriate. Data are presented as mean ± standard deviation (SD). Statistical significance was indicated using asterisks (* *p* < 0.05, ** *p* < 0.01, and *** *p* < 0.001). 

## 5. Conclusions

This study reframes muscle-stage *T. spiralis* larvae as experimentally accessible yet biologically constrained drug targets. It also introduces a validated metabolic assay for quantitative interrogation of this stage. While TBD demonstrates robust activity within this framework, its broader value lies in illustrating how alternative mechanisms of action can be systematically evaluated against a historically refractory parasite stage. Future studies leveraging this platform to optimize dosing strategies and explore rational drug combinations may further advance therapeutic development for trichinellosis.

## Figures and Tables

**Table 1:** Inhibitory concentration 50 (IC_50_) of TBD, IVM, MEQ and ABZ as determined by XTT and microscopy (µM).

Drugs	Microscopy	95% CI	XTT	95% CI
Albendazole	1.550	1210–2260	543.6	Very wide
Tribendimine	135.2	123.8–148.2	208.6	189.9–227.1
Ivermectin	349.9	309.1–400.9	320.0	293.0–348.1
Mefloquine	414.9	382.3–455.0	431.7	350.9–657.9

**Table 2:** Reductions of *T. spiralis* larvae in the diaphragm and gastrocnemius of mice after treatment with TBD and ABZ.

Group	Diaphragm	Gastrocnemius
Inf. Control	6169 ± 1921	989 ± 295.7
TBD	799 ± 199.4 ** (87.0%)	265.0 ± 53.2 ** (73.2%)
ABZ	844 ± 184.6 ** (86.3%)	259.5 ± 77.5 ** (73.8%)

## Data Availability

The original contributions presented in this study are included in the article. Further inquiries can be directed at the corresponding author.

## Abbreviations

The following abbreviations are used in this manuscript:

ABZ	albendazole
MLT	miltefosine
IVM	ivermectin
TBD	tribendimidine
PZQ	praziquantel
ART	artesunate
MEQ	mefloquine
XTT	sodium salt 2,3-Bis(2-methoxy-4-nitro-5-sulfophenyl)-2H-tetrazolium-5-carboxanilide

## References

[1] Dupouy-Camet J. (2000). Trichinellosis: Aworldwide zoonosis. Vet. Parasitol..

[2] Gottstein B., Pozio E., Nöckler K. (2009). Epidemiology, diagnosis, treatment, and control of trichinellosis. Clin. Microbiol. Rev..

[3] Murrell K.D., Pozio E. (2011). Worldwide occurrence and impact of human trichinellosis, 1986–2009. Emerg. Infect. Dis..

[4] Cuperlovic K., Djordjevic M., Pavlovic S. (2005). Re-emergence of trichinellosis in southeastern Europe due to political and economic changes. Vet. Parasitol..

[5] Rostami A., Gamble H.R., Dupouy-Camet J., Khazan H., Bruschi F. (2017). Meat sources of infection for outbreaks of human trichinellosis. Food Microbiol..

[6] Bruschi J. (2014). Helminth Infections and Their Impact on Global Public Health.

[7] Daniel-Mwambete K., Torrado S., Cuesta-Bandera C., Ponce-Gordo F., Torrado J. (2004). The effect of solubilization on the oral bioavailability of three benzimidazole carbamate drugs. Int. J. Pharm..

[8] Yadav A.K. (2012). Temjenmongla Efficacy of *Lasia spinosa* leaf extract in treating mice infected with *Trichinella spiralis*. Parasitol. Res..

[9] Prichard R.K. (2007). Markers for benzimidazole resistance in human parasitic nematodes. Parasitology.

[10] Abuelenain G.L., Fahmy Z.H., Elshennawy A.M., Selim E.H.A., Elhakeem M., Hassanein K.M.A., Awad S.M. (2022). Phenotypic Changes of *Trichinella spiralis* Treated By *Commiphora molmol*, *Lepidium sativum*, and Albendazole: In Vitro Study. Helminthologia.

[11] El-Sayad M.H., El-Wakil E.S., Moharam Z.H., Abd El-Latif N.F., Ghareeb M.A., Elhadad H. (2023). Repurposing drugs to treat trichinellosis: Invitroanalysis of the anthelmintic activity of nifedipine and *Chrysanthemum coronarium* extract. BMC Complement. Med. Ther..

[12] Katiyar J.C., Gupta S., Sharma S. (1989). Experimental models in drug development for helminthic diseases. Rev. Infect. Dis..

[13] Kotze A.C. (2012). Target-based and whole-worm screening approaches to anthelmintic discovery. Vet. Parasitol..

[14] Stevens M.G., Olsen S.C. (1993). Comparative analysis of using MTT and XTT in colorimetric assays for quantitating bovine neutrophil bactericidal activity. J. Immunol. Methods.

[15] Meshulam T., Levitz S.M., Christin L., Diamond R.D. (1995). A simplified new assay for assessment of fungal cell damage with the tetrazolium dye, (2,3)-bis-(2-methoxy-4-nitro-5-sulphenyl)-(2H)-tetrazolium-5-carboxanilide (XTT). J. Infect. Dis..

[16] Williams C., Espinosa O.A., Montenegro H., Cubilla L., Capson T.L., Ortega-Barría E., Romero L.I. (2003). Hydrosoluble formazan XTT: Its application to natural products drug discovery for Leishmania. J. Microbiol. Methods.

[17] Aguiar P.H.N., Fernandes N.M.G.S., Zani C.L., Mourão M.M. (2017). A high-throughput colorimetric assay for detection of *Schistosoma mansoni* viability based on the tetrazolium salt XTT. Parasites Vectors.

[18] Lee S.O., Chu K.B., Yoon K.W., Heo S.I., Song J.H., Li J., Hong S.J., Quan F.S. (2024). Combinatorial Treatment with Praziquantel and Curcumin Reduces *Clonorchis sinensis* Parasite Burden and Clonorchiasis-Associated Pathologies in Rats. Pharmaceutics.

[19] Muñoz-Carrillo J.L., Muñoz-Escobedo J.J., Maldonado-Tapia C.H., Chávez-Ruvalcaba F., Moreno-García M.A. (2017). Resiniferatoxin lowers TNF-α, NO and PGE2 in the intestinal phase and the parasite burden in the muscular phase of *Trichinella spiralis* infection. Parasite Immunol..

[20] El-Wakil E.S., Khodear G.A.M., Ahmed H.E.S., Ibrahim G.I.K., Hegab F., Abdo S.M. (2023). Therapeutic efficacy of albendazole and berberine loaded on bovine serum albumin nanoparticles on intestinal and muscular phases of experimental trichinellosis. Acta Trop..

[21] Allam A.F., Mostafa R.A., Lotfy W., Farag H.F., Fathi N., Moneer E.A., Shehab A.Y. (2021). Therapeutic efficacy of mebendazole and artemisinin in different phases of trichinellosis: A comparative experimental study. Parasitology.

[22] Fahmy A.M., Diab T.M. (2021). Therapeutic Efficacy of Albendazole and Mefloquine Alone or in Combination Against Early and Late Stages of *Trichinella spiralis* Infection in Mice. Helminthologia.

[23] Chung M.S., Joo K.H., Quan F.S., Kwon H.S., Cho S.W. (2001). Efficacy of flubendazole and albendazole against *Trichinella spiralis* in mice. Parasite.

[24] Buchter V., Priotti J., Leonardi D., Lamas M.C., Keiser J. (2020). Preparation, Physicochemical Characterization and In Vitro and In Vivo Activity Against *Heligmosomoides polygyrus* of Novel Oral Formulations of Albendazole and Mebendazole. J. Pharm. Sci..

[25] Yue W.W., Yan S.W., Zhang R., Cheng Y.K., Liu R.D., Long S.R., Zhang X., Wang Z.Q., Cui J. (2022). Characterization of a novel pyruvate kinase from *Trichinella spiralis* and its participation insugarmetabolism, larval molting and development. PLoS Neglected Trop. Dis..

[26] Yao C., Jasmer D.P. (2001). Trichinella spiralis-infected muscle cells: Abundant RNA polymerase II in nuclear speckle domains colocalizes with nuclear antigens. Infect. Immun..

[27] Sánchez-Montejo J., Marín M., Villamizar-Monsalve M.A., Vieira M.D.C., Vicente B., Peláez R., López-Abán J., Muro A. (2025). AxiWorm: A new tool using YOLOv5 to test antiparasitic drugs against *Trichinella spiralis*. Parasites Vectors.

[28] Basyoni M.M., El-Sabaa A.A. (2013). Korean Therapeutic potential of myrrh and ivermectin against experimental *Trichinella spiralis* infection in mice. Kor. J. Parasitol..

[29] Soliman G.A., Taher E.S., Mahmoud M.A. (2011). Therapeutic efficacy of Dormectin, Ivermectin and Levamisole against different stages of *Trichinella spiralis* in rats. Turk. Parazitolojii Derg..

[30] Hu Y., Xiao S.H., Aroian R.V. (2009). The new anthelmintic tribendimidineis an L-type (levamisole and pyrantel) nicotinic acetylcholine receptor agonist. PLoS Neglected Trop. Dis..

